# Marine Bioactives in Liver Aging: Mechanistic Insights and Translational Potential

**DOI:** 10.3390/md24040138

**Published:** 2026-04-15

**Authors:** Ricardo Moreno Traspas, Zachariah Tman

**Affiliations:** 1 Independent Researcher, San Diego, CA 92122, USA; 2 Independent Researcher, Philadelphia, PA 19122, USA

**Keywords:** liver, aging, marine bioactives, rejuvenation, senescence, oxidative stress, metabolic dysfunction, epigenetics

## Abstract

The liver is a central regulator of systemic metabolism and exhibits exceptional regenerative capacity, yet aging progressively impairs hepatic resilience through metabolic dysregulation, mitochondrial dysfunction, epigenetic instability, and chronic inflammation. Marine ecosystems constitute a vast and underexplored source of structurally diverse bioactive compounds that have evolved to modulate conserved stress response and homeostatic pathways. This review synthesizes current preclinical evidence demonstrating how marine-derived metabolites target key molecular axes implicated in liver aging, including energy sensing, redox balance, mitochondrial quality control, inflammatory signaling, and chromatin-associated regulation. Rather than focusing solely on isolated hepatoprotective effects, we frame marine bioactives within an aging biology perspective, highlighting their ability to modulate pathways associated with cellular plasticity and resilience. We further propose that this mechanistic convergence provides a theoretical framework for exploring marine compounds as potential adjunctive modulators within emerging, experimental liver rejuvenation strategies, including partial cellular reprogramming approaches that require coordinated metabolic and epigenetic control. While acknowledging that direct reversal of liver aging remains to be clinically established, integrating marine chemodiversity with contemporary aging and regenerative biology outlines a conceptual roadmap for developing liver-directed interventions targeting aging-related vulnerability as a fundamental driver of disease.

## 1. Introduction

The liver is the central metabolic engine of the human body and one of the most functionally complex organs in human physiology. Acting as a master regulator of systemic homeostasis, it integrates nutrient metabolism, endocrine signaling, and energy balance to maintain metabolic stability, systemic detoxification capacity, and immune equilibrium across the lifespan [1,2,3]. Hepatocytes, in coordination with Kupffer cells, hepatic stellate cells, sinusoidal endothelial cells, and resident immune populations, orchestrate a vast array of biological processes. These processes encompass nutrient metabolism, detoxification, bile production, and the synthesis of plasma proteins and signaling factors essential for vascular and immune homeostasis [2,3,4,5].

Despite its remarkable regenerative capacity, the liver is not immune to biological aging. Progressive hepatocellular aging is characterized by the accumulation of senescent cells, mitochondrial dysfunction, redox imbalance, epigenetic drift, and metabolic inflexibility [6,7]. Cellular senescence leads to a durable cell-cycle arrest and the development of a senescence-associated secretory phenotype (SASP), which promotes chronic low-grade inflammation and maladaptive tissue remodeling [8,9,10]. Hepatocyte senescence can also propagate systemically, contributing to extrahepatic multi-organ dysfunction independently of chronological aging [11]. In parallel, age-associated mitochondrial decline increases reactive oxygen species (ROS) production driving oxidative damage, while also impairing ATP generation and metabolic efficiency [12,13,14,15]. These processes are accompanied by dysregulation of nutrient-sensing pathways, altered lipid and glucose metabolism, and reduced regenerative responsiveness [6,7]. Together, these hallmarks of aging progressively reshape the hepatic microenvironment and reduce resilience to injury. Over time, the liver shifts from a highly regenerative organ to one increasingly vulnerable to chronic damage, fibrosis, and malignant transformation.

**Figure 1:**
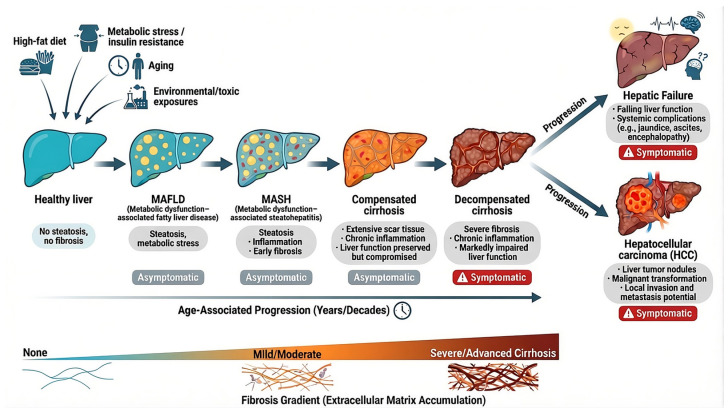
Aging-associated progression of metabolic dysfunction-related liver disease. Schematic illustrating the sequential stages of liver disease, initiated by a combination of aging, metabolic stress, diet, and toxic exposures. The disease progresses from metabolic dysfunction-associated steatotic liver disease (MAFLD) to metabolic dysfunction-associated steatohepatitis (MASH), compensated cirrhosis, decompensated cirrhosis, hepatic failure, and hepatocellular carcinoma (HCC). Early stages (MAFLD, MASH, compensated cirrhosis) are largely asymptomatic, whereas advanced stages (decompensated cirrhosis, hepatic failure, HCC) are symptomatic. Fibrosis, chronic inflammation, and impaired hepatic function increase with both disease progression and aging.

Clinically, these molecular and cellular changes manifest as a progressive spectrum of chronic liver diseases that rise sharply with age (Figure 1). While genetic predisposition and disease-specific risk alleles contribute to individual susceptibility [16,17,18,19], the initiation and progression of most chronic liver diseases are predominantly driven by the cumulative acquisition of aging hallmarks and the lifelong impact of environmental insults [20,21] (Figure 1). Metabolic dysfunction-associated steatotic liver disease (MAFLD) and its inflammatory subtype, metabolic dysfunction-associated steatohepatitis (MASH), represent the hepatic expression of systemic metabolic syndrome (Figure 1). Lipid accumulation, insulin resistance, mitochondrial stress, and oxidative injury create a permissive environment for inflammation, hepatocyte death, and fibrogenic signaling [22,23].

Persistent hepatic injury, regardless of etiology, activates maladaptive wound-healing responses that drive fibrogenesis [24,25] (Figure 1). Hepatic stellate cells transdifferentiate into myofibroblast-like cells, producing excessive extracellular matrix components progressively replacing functional parenchyma with fibrotic scar tissue [26,27]. While early-stage fibrosis is partially reversible, sustained injury often leads to cirrhosis, characterized by architectural distortion, chronic inflammation, portal hypertension, and progressive hepatic insufficiency [28] (Figure 1). Cirrhosis represents the terminal common pathway of most chronic liver diseases and remains the dominant precursor to hepatic failure and hepatocellular carcinoma [29] (Figure 1). The aged cirrhotic liver provides a pro-oncogenic landscape defined by genomic instability, telomere attrition, epigenetic dysregulation, and persistent inflammatory signaling, creating fertile ground for malignant transformation [30].

The societal and economic burden of these conditions is substantial and growing. Liver disease remains among the leading global causes of mortality, with cirrhosis accounting for over one million deaths annually and contributing significantly to global disability-adjusted life years (DALYs) [31,32]. Notably, MASH is frequently underdiagnosed, as early disease is often clinically silent and lacks reliable noninvasive diagnostic markers (Figure 1). Population-based estimates suggest a prevalence of approximately 3–5% among adults, with up to 80–85% of affected individuals remaining undiagnosed [33]. Consequently, many patients are only diagnosed at advanced stages, such as established fibrosis or decompensated cirrhosis, when therapeutic options are limited (Figure 1). Despite major advances in etiological management, current clinical approaches remain largely palliative. For patients with decompensated cirrhosis, liver transplantation remains the only curative option, yet its impact is constrained by donor shortages, high costs, immunosuppression complications, and limited access [34,35]. This therapeutic stagnation underscores a fundamental gap in hepatology: the absence of strategies capable of reversing fibrosis and restoring biological youthfulness and functional resilience to the aging liver, rather than merely slowing its decline.

In response, the concept of liver rejuvenation has emerged as a paradigm shift that reframes aging itself as a modifiable driver of hepatic dysfunction. Advances in aging biology and regenerative medicine demonstrate that cellular aging is a plastic biological state that can be partially reset. Central to this insight is partial cellular reprogramming, in which transient perturbation of core transcriptional regulators restores youthful gene expression patterns and metabolic competence without erasing somatic identity [36,37,38,39]. In parallel, non-genetic chemical reprogramming strategies target conserved aging networks—including chromatin modifiers, mitochondrial function, and nutrient-sensing pathways—to achieve functional rejuvenation through pharmacological means [40,41].

Despite their promise, liver rejuvenation strategies remain at an early translational stage. Safe delivery, durability of effect, tissue specificity, and precise control of reprogramming intensity continue to limit clinical applicability [39]. Critically, the field lacks a sufficiently diverse and biologically compatible repertoire of small molecules capable of modulating aging hallmarks in a controlled, hepatocyte-centered manner. This gap underscores the need for new molecular scaffolds and mechanistic modalities that can interface with hepatic metabolism, redox biology, epigenetic regulation, and fibrotic signaling.

Against this backdrop, the marine environment represents a compelling and still underexplored source of bioactive molecules for liver research. Marine organisms have evolved under extreme and fluctuating physicochemical conditions, including hypoxia, osmotic stress, and intense ecological competition, driving the emergence of structurally unique secondary metabolites rarely found in terrestrial systems [42]. The translational relevance of this chemical diversity is supported by the successful development of several marine-derived therapeutics, particularly in oncology and infectious diseases [43,44]. In the context of liver biology, growing preclinical evidence indicates that marine natural products can modulate key convergent processes implicated in age-associated liver dysfunction, including redox homeostasis, mitochondrial function, metabolic regulation, and fibrotic remodeling.

Experimental strategies aimed at liver rejuvenation thus represent a rapidly emerging but still immature field, in which the need for novel molecular frameworks, safer modulators of aging pathways, and biologically integrated therapeutic strategies remains substantial. The convergence of marine chemodiversity with modern concepts in aging and regenerative biology offers a rational and promising avenue to address this gap. This framework positions marine-derived bioactives not merely as hepatoprotective agents, but as potential pathway-directed modulators of hepatic aging itself, pending clinical validation.

Several review articles have previously examined the hepatoprotective effects of marine-derived bioactives, primarily within the context of specific liver diseases and canonical pathogenic mechanisms [45,46,47]. However, these studies largely adopt a disease-centered perspective and do not explicitly consider liver aging as a unifying biological framework. Here, we instead integrate marine bioactives within the context of aging biology, focusing on their preclinical interactions with key molecular hallmarks of liver aging, including metabolic dysregulation, mitochondrial dysfunction, oxidative stress, epigenetic alterations, and cellular senescence. This perspective enables a more cohesive understanding of how these compounds may influence hepatic function and resilience, particularly in experimental models of age-associated conditions such as MASH and liver fibrosis.

This review is not intended to be systematic; however, a structured literature selection approach was employed using major scientific databases, including PubMed, Web of Science, and Scopus, to ensure conceptual coherence and relevance. Studies were included based on their focus on marine-derived bioactive compounds and their ability to provide mechanistic insights into pathways associated with liver aging and/or evidence from preclinical models of age-related liver diseases, including MASH and fibrosis. Emphasis was placed on recent studies, primarily those published within the last decade, elucidating conserved aging-related processes rather than solely descriptive hepatoprotective effects. Together, this approach enables a focused synthesis of current knowledge while highlighting emerging hypotheses and preclinical connections between marine chemodiversity and liver aging biology.

## 2. Marine Bioactives Targeting Oxidative Stress and Senescence in Liver Cells

### 2.1. The Redox–Senescence Axis as a Central Driver of Hepatic Functional Decline (Figure 2)

Liver aging is driven by a self-amplifying feedback loop between oxidative stress and cellular senescence. As the body’s central metabolic and detoxification hub, the liver sustains exceptionally high oxygen consumption and harbors a dense mitochondrial network, rendering hepatocytes a primary site for continuous ROS generation. In young and metabolically resilient livers, redox homeostasis is preserved through robust antioxidant defenses, efficient mitochondrial quality control, and adaptive stress response signaling. With advancing age, however, this balance progressively deteriorates, giving rise to a characteristic “redox drift” toward a pro-oxidant state [12,13,14,15]. This shift is driven by age-associated mitochondrial dysfunction, impaired electron transport chain efficiency, and defective mitophagy, which together promote electron leakage and sustained superoxide production [12,13,14,15]. Additional hepatic sources of oxidative stress include peroxisomes, cytochrome P450 monooxygenases, and NADPH oxidases [48]. The convergence of these ROS sources creates a chronic oxidative milieu that exceeds the buffering capacity of aging hepatocytes.

To counteract this oxidative burden, liver cells rely on a multilayered antioxidant network encompassing enzymatic and non-enzymatic systems. Central to the coordination of these defenses is Nuclear factor erythroid 2-related factor 2 (NRF2), the master regulator of cellular redox homeostasis [49]. Under basal conditions, NRF2 is sequestered in the cytoplasm by its inhibitor Kelch-like ECH-associated protein 1 (KEAP1) and targeted for proteasomal degradation. Oxidative or electrophilic stress modifies key cysteine residues on KEAP1, allowing NRF2 to escape degradation, translocate to the nucleus, and activate antioxidant response element (ARE)-driven transcriptional programs. These programs encompass antioxidant enzymes, phase II detoxification machinery, and metabolic stress response proteins essential for hepatocyte resilience [49]. However, NRF2 signaling efficiency declines with age. Persistent metabolic overload, chronic inflammation, and altered redox signaling blunt NRF2 responsiveness, weakening antioxidant capacity precisely as ROS production escalates [50,51,52]. As a result, oxidative stress transitions from an adaptive signaling cue into a sustained driver of cellular dysfunction.

At the cellular level, chronic oxidative stress serves as a primary trigger for hepatocellular senescence—a stable state of irreversible growth arrest accompanied by profound functional remodeling. ROS-induced genomic instability, mitochondrial distress, and accelerated telomere attrition converge on canonical senescence pathways governed by the p53/p21^WAF1^ and p16^INK4A^/Rb axes [9,10,13,14,53]. Senescent hepatocytes accumulate in proportion to disease severity, comprising up to 70–80% of parenchymal cells in advanced cirrhosis [54]. Importantly, senescent burden correlates strongly with fibrosis severity, hepatic decompensation, and overall mortality, underscoring senescence as a causal determinant of liver functional decline rather than a passive epiphenomenon [55].

A defining feature of senescent hepatocytes is the acquisition of the SASP. Through the SASP, senescent cells secrete a complex mixture of pro-inflammatory cytokines, chemokines, growth factors, and matrix-remodeling enzymes [9,10,13,14,53]. This secretome exerts potent autocrine and paracrine effects, propagating senescence to neighboring cells and sustaining chronic low-grade inflammation, a process often termed “inflammaging” [56]. SASP mediators directly activate hepatic stellate cells, promoting their transdifferentiation into collagen-producing myofibroblasts and driving progressive extracellular matrix deposition [57,58]. In this context, the redox–senescence axis functions as a mechanistic bridge linking physiological aging to pathological fibrosis.

Mitochondrial dysfunction lies at the core of this persistence. Loss of mitochondrial membrane integrity in senescent hepatocytes facilitates cytosolic release of mitochondrial DNA, which activates innate immune pathways such as the cGAS–STING axis [9,10,59]. This coupling provides a direct molecular link between oxidative stress and sterile inflammation, reinforcing SASP expression and amplifying tissue injury. In parallel, senescent hepatocytes upregulate pro-survival pathways that confer resistance to apoptosis, allowing these dysfunctional cells to evade immune-mediated clearance and persist within the hepatic parenchyma [9,10,60].

Beyond structural remodeling and inflammation, the redox–senescence axis imposes a substantial metabolic penalty. Senescent hepatocytes exhibit marked metabolic inflexibility, characterized by impaired β-oxidation, disrupted NAD^+^ homeostasis, and dysregulation of nutrient-sensing pathways [9,10,54,61]. These defects translate into diminished detoxification capacity and compromised bile synthesis and transport. Downregulation of key canalicular transporters, including MRP2 and MRP3, increases susceptibility to cholestasis and drug-induced liver injury in aging populations [62].

Collectively, these interconnected processes define a self-reinforcing pathological circuit in which mitochondrial dysfunction drives excess ROS production, oxidative stress induces senescence, and senescent cells amplify inflammatory and fibrotic signaling (Figure 3). Once entrenched, this circuit establishes a functional “point of no return” under conventional therapeutic paradigms. Effective intervention therefore requires coordinated modulation of multiple nodes within the redox–senescence axis. As explored in the following sections, the marine pharmacopeia represents a uniquely rich source of bioactives capable of engaging these nodes simultaneously, emerging as promising candidates for restoring hepatic resilience during aging.

**Figure 2:**
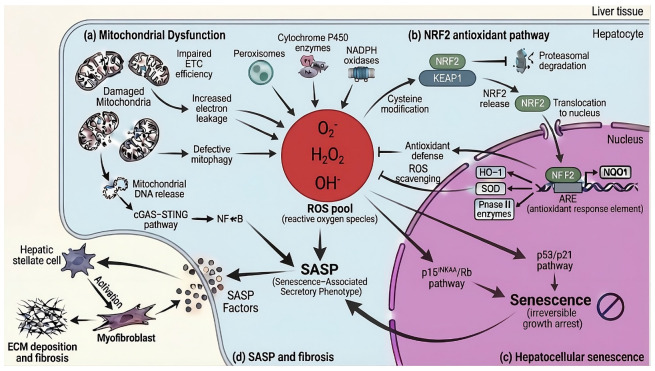
Redox–senescence axis driving liver aging and fibrosis. (**a**) Age-associated mitochondrial dysfunction, characterized by impaired electron transport and defective mitophagy, alongside peroxisomes and cytosolic enzymes, drives excess reactive oxygen species (ROS) generation. In parallel, cytosolic leakage of damaged mitochondrial DNA activates the innate immune cGAS-STING/NF-κB axis. (**b**) Activation of the NRF2 antioxidant pathway coordinates adaptive transcriptional responses to oxidative stress but becomes insufficient with persistent insult. (**c**) Sustained redox imbalance and mitochondrial stress induce hepatocellular senescence through canonical cell-cycle arrest pathways. (**d**) Senescent hepatocytes adopt a senescence-associated secretory phenotype (SASP), releasing factors that reinforce the inflammatory niche and activate hepatic stellate cells to transdifferentiate into myofibroblasts, progressively driving fibrogenesis. ETC: electron transport chain. ECM: extracellular matrix.

**Figure 3:**
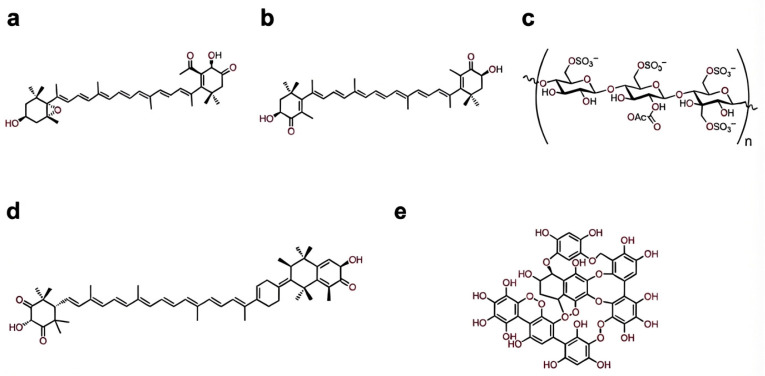
Representative marine bioactives discussed in this review. Chemical structures of selected marine-derived compounds with documented hepatoprotective and metabolic regulatory activity. The unique chemical architectures of these molecules, including specialized functional groups, polarity, and charge density, dictate their distinct mechanisms of action within the hepatic microenvironment: (**a**) fucoxanthin, (**b**) astaxanthin, (**c**) fucoidan, (**d**) siphonaxanthin, and (**e**) dieckol.

### 2.2. Fucoxanthin

Fucoxanthin is a marine xanthophyll carotenoid produced predominantly by brown macroalgae and photosynthetic microalgae, where it functions as an accessory pigment within light-harvesting complexes [63]. Structurally distinct from most dietary carotenoids, fucoxanthin incorporates an allenic bond, a 5,6-monoepoxide, a conjugated carbonyl group, and an acetylated terminal moiety [64,65] (Figure 3a). The rare allenic bond introduces axial chirality into the polyene backbone and contributes to enhanced chemical reactivity [66] (Figure 3a). These features underpin fucoxanthin’s dual antioxidant behavior, combining efficient physical quenching of singlet oxygen via its extended conjugated system with direct radical scavenging mediated by its polar functional groups [67] (Figure 3a). This combination allows fucoxanthin to buffer oxidative stress without rapid molecular depletion, a property particularly relevant in metabolically active tissues such as the liver.

Although the overall systemic absorption of native fucoxanthin is low, it functions as a highly effective pro-bioactive whose pharmacokinetic journey is defined by sequential, tissue-specific biotransformation. Following oral intake, intestinal esterases rapidly deacetylate native fucoxanthin into fucoxanthinol, the primary circulating metabolite [68,69]. Upon reaching the hepatic microenvironment, the liver acts as both a primary bioaccumulation site and an active metabolic hub. Fucoxanthinol achieves its highest tissue area-under-the-curve (AUC) here, where it undergoes targeted, NADP^+^-dependent dehydrogenation to generate amarouciaxanthin A [68,69,70]. Following this liver-targeted metabolism, amarouciaxanthin A preferentially partitions into adipose depots [70]. The identification of additional intermediates, such as halocynthiaxanthin, further supports that the liver’s active metabolic processing is essential to expanding fucoxanthin’s functional spectrum and translational potential [63].

At the cellular level, fucoxanthin and its metabolites act as pleiotropic regulators of redox homeostasis and stress adaptation pathways that deteriorate during hepatocyte aging. A central component of this activity is activation of the NRF2-dependent antioxidant response. Fucoxanthin promotes dissociation of NRF2 from KEAP1, facilitating nuclear translocation and induction of cytoprotective genes involved in antioxidant defense and detoxification [71,72]. This response is supported by increased SIRT1 activity, which enhances NRF2 deacetylation and stabilizes its transcriptional output [73,74]. The resulting upregulation of phase II enzymes, including heme oxygenase-1 (HO-1), superoxide dismutase (SOD), and NAD(P)H quinone dehydrogenase 1 (NQO1), effectively limits intracellular ROS accumulation and preserves genomic integrity under chronic metabolic stress [71,72].

Beyond cytosolic redox control, fucoxanthin modulates mitochondrial quality control processes that become progressively compromised with age. Activation of AMP-activated protein kinase (AMPK) enhances mitochondrial stress resilience, helping preserve membrane potential and constrain mitochondrial-derived ROS production [75]. These effects are consistent with improved clearance of damaged mitochondria and maintenance of energetic stability [75]. Emerging evidence further suggests that fucoxanthin may influence mitochondrial calcium handling and uncoupling dynamicsin metabolically stressed hepatocytes, although these mechanisms require further validation [76,77].

By constraining sustained oxidative stress and mitochondrial dysfunction, fucoxanthin indirectly suppresses activation of the DNA damage response and downstream senescence programs. This is reflected by attenuation of p53/p21 and p16^INK4A^ signaling pathways, reducing the likelihood of irreversible cell-cycle arrest [78,79]. In parallel, specifically in hepatocytes fucoxanthin dampens inflammatory reinforcement loops by inhibiting the TLR4/NF-κB axis, leading to reduced expression of pro-inflammatory cytokines such as TNF-α, IL-6, and IL-1β [72,80,81]. This suppression of the SASP limits paracrine propagation of senescence within the hepatic microenvironment. Collectively, these properties position fucoxanthin as a chemically privileged marine carotenoid capable of engaging multiple nodes of the redox–senescence axis to preserve liver homeostasis (Table 1).

### 2.3. Astaxanthin

Astaxanthin (3,3′-dihydroxy-β,β′-carotene-4,4′-dione) is a marine-derived xanthophyll carotenoid synthesized primarily by the microalga *Haematococcus pluvialis* [82,83]. Its distinctive molecular architecture, featuring polar ionone rings flanking a long conjugated polyene chain, enables astaxanthin to span lipid bilayers with defined orientation [82,83] (Figure 3b). This transmembrane configuration allows simultaneous interception of ROS at both the hydrophobic core and hydrophilic interface of cellular membranes, conferring antioxidant potency that exceeds that of structurally simpler carotenoids and classical lipophilic antioxidants [82,83]. Following oral administration, unmodified astaxanthin exhibits low systemic bioavailability driven by a combination of high lipophilicity, poor gastrointestinal absorption, and a massive gastrointestinal first-pass effect (~90% extraction) [84,85]. However, particularly when coupled with specific delivery systems to enhance gut uptake, astaxanthin demonstrates a strong hepatic tissue affinity [86,87]. Upon reaching the hepatic compartment, astaxanthin undergoes targeted, saturable metabolism driven primarily by hepatic cytochrome P450 enzymes, notably the CYP1A1/2 isoforms [85,88].

In hepatocytes, astaxanthin functions as a potent mitoprotective agent, counteracting oxidative stress-induced mitochondrial dysfunction. By stabilizing mitochondrial membranes and limiting lipid peroxidation, astaxanthin preserves mitochondrial membrane potential, suppresses excessive mitochondrial ROS generation, and constrains vulnerability to ferroptosis—an iron-dependent form of regulated cell death increasingly implicated in liver aging and chronic liver disease [89,90,91]. In aging hepatocytes, astaxanthin engages the NRF2/HO-1 axis in a context-dependent manner, preferentially supporting glutathione redox buffering and iron-sensitive lipid peroxide detoxification [90,91]. In parallel, astaxanthin increases expression of autophagy machinery, and influences mitophagy pathways associated with mitochondrial quality control, which may help prevent accumulation of damaged organelles and oxidative stress-related cellular dysfunction [92,93,94,95].

At the nuclear level, astaxanthin suppresses activation of senescence-associated cell-cycle inhibitors, including p16^INK4A^, thereby reducing the probability of irreversible growth arrest [96]. Through inhibition of NF-κB and MAPK signaling, astaxanthin dampens SASP expression, reducing secretion of pro-inflammatory mediators such as IL-1β, IL-6, and TNF-α [97,98,99]. Attenuation of this inflammatory secretome, together with reduced TGF-β1 signaling, limits hepatic stellate cell activation and constrains the development of a pro-senescent fibrotic niche [100,101,102]. Collectively, these effects position astaxanthin as a multifunctional marine bioactive that integrates membrane-level antioxidant defense, mitochondrial preservation, and suppression of senescence-promoting inflammatory circuits (Table 1).

### 2.4. Fucoidan

Fucoidan is a sulfated marine polysaccharide predominantly isolated from brown macroalgae, characterized by a heterogeneous and branched backbone composed mainly of α-L-fucopyranose units linked through (1→3) and (1→4) glycosidic bonds, with variable incorporation of other sugars like galactose, mannose, and uronic acids [103,104,105] (Figure 3c). Its biological activity is tightly coupled to structural parameters including molecular weight, degree and pattern of sulfation, and accessory monosaccharides, which influence interactions with biological targets [103,104,105] (Figure 3c). The high negative charge density conferred by abundant sulfate groups enables fucoidan to function as a polyanionic glycosaminoglycan mimetic in biological systems, facilitating interactions with redox-sensitive receptors and signaling complexes at the hepatocyte surface and in immune cells [106,107] (Figure 3c). Despite its high molecular weight and dense negative charge severely limiting the absolute systemic bioavailability of oral fucoidan, pharmacokinetic mapping reveals that absorbed fucoidan preferentially accumulates in the kidneys, spleen and liver, where it specifically localizes within sinusoidal non-parenchymal populations, including Kupffer cells [108,109].

In hepatocytes, fucoidan mitigates oxidative stress-driven senescence primarily through activation of the NRF2-dependent antioxidant response. Fucoidan promotes stabilization and nuclear accumulation of NRF2, leading to selective induction of cytoprotective genes involved in redox buffering and detoxification [107,110,111,112]. Rather than broadly amplifying antioxidant enzyme expression, fucoidan-driven NRF2 signaling preferentially enhances HO-1, NQO1, and glutathione biosynthetic pathways, thereby sustaining intracellular redox balance under chronic oxidative flux [107,110,111,112].

By constraining oxidative damage, fucoidan indirectly suppresses activation of the DNA damage response, a central trigger of senescence entry. Experimental models indicate that fucoidan modulates key senescence-associated cell-cycle regulators, including p21^WAF1^/Cip1 and p16^INK4A^, thereby limiting the establishment of irreversible growth arrest in stressed hepatocytes [113]. In parallel, fucoidan exerts robust control over inflammatory reinforcement loops by inhibiting NF-κB nuclear translocation and stress-activated MAPK signaling, resulting in marked suppression of the SASP [107,114]. Reduced secretion of pro-inflammatory mediators, together with interference in TGF-β1/Smad signaling, prevents establishment of a pro-fibrotic, pro-senescent niche [115,116,117]. Through coordinated modulation of redox homeostasis, DNA damage signaling, and inflammatory amplification, fucoidan emerges as a structurally complex marine polysaccharide capable of preserving hepatocyte functional competence during aging (Table 1).

### 2.5. Bromophenols and Marine Peptides

Beyond carotenoids and polysaccharides, marine bromophenols and bioactive peptides represent structurally and functionally distinct classes of compounds that converge on the redox–senescence axis through complementary mechanisms. Their chemical diversity enables coordinated modulation of oxidative stress initiation, mitochondrial resilience, and inflammatory reinforcement loops that collectively drive hepatic aging.

Marine bromophenols, particularly those isolated from red algae of the *Rhodomelaceae* family, are defined by polyphenolic scaffolds densely substituted with bromine atoms [118,119]. This extensive halogenation enhances electrophilicity and confers heightened reactivity toward redox-sensitive cysteine residues within regulatory proteins [118,119]. Bromophenols such as bis(2,3-dibromo-4,5-dihydroxybenzyl) ether (BDDE) act as non-canonical activators of the NRF2–ARE pathway by disrupting KEAP1-mediated repression, thereby inducing robust transcription of cytoprotective genes [120,121]. Several bromophenol derivatives additionally inhibit protein tyrosine phosphatase 1B (PTP1B), a negative regulator of insulin signaling increasingly linked to metabolic stress-driven oxidative damage in the aging liver [122,123]. Through the coordinated modulation of redox defense and insulin sensitivity, bromophenols preserve hepatic metabolic flexibility [124].

Marine-derived bioactive peptides provide a complementary, downstream layer of protection against hepatocyte oxidative stress and senescence. These peptides are generated through controlled enzymatic hydrolysis of marine proteins derived from sources including fish collagen, mollusks, and microalgae. Typically composed of 2–10 amino acids and generally <1000 Da, marine peptides display favorable bioavailability and tissue penetration compared with intact proteins [125,126]. Representative sequences such as the fish-derived dipeptide Ala–Tyr (AY) and the heptapeptide Asp–Leu–Val–Lys–Val–Glu–Ala isolated from *Lycodes diapterus* have been shown to exert antioxidant and cytoprotective effects [127,128]. In hepatocytes, marine oligopeptides such as QDYD, ARW, and YPAGP isolated from monkfish act primarily as antioxidant regulators and mitochondrial stabilizers [129]. In models of lipid-induced stress, these peptides mitigate oxidative stress and lipid accumulation by activating the AMPK/NRF2 signaling axis, which suppresses lipogenesis while promoting fatty acid β-oxidation [129]. NRF2 activation also enhances the expression of HO-1 and endogenous antioxidant enzymes, effectively scavenging intracellular ROS and preserving mitochondrial function [129].

Together, bromophenols and marine peptides provide a multi-tiered defense against hepatocyte aging (Table 1). Acting at distinct but convergent nodes of the redox–senescence axis, these compounds reinforce hepatic resilience and complement broader marine-derived strategies aimed at mitigating age-associated liver dysfunction (Table 1).

## 3. Marine Compounds as Modulators of Metabolic and Epigenetic Pathways in Liver Aging

### 3.1. Metabolic Decline and Epigenetic Drift in the Aging Liver (Figure 4)

Aging of the liver is characterized by a progressive uncoupling of cellular energy status from genomic regulation. Central to this process is depletion of the hepatic NAD^+^ pool, which destabilizes the SIRT1–AMPK–PGC-1α axis—a conserved regulatory network integrating nutrient sensing with chromatin remodeling, mitochondrial maintenance, and adaptive gene expression [130,131,132,133]. Under youthful conditions, this axis functions as a tightly coordinated feedback system, allowing energetic stress to be efficiently transduced into transcriptional and epigenetic programs that preserve metabolic flexibility and cellular identity. With advancing age, however, increased activity of NAD^+^-consuming enzymes, particularly CD38 and PARP1, progressively diverts substrate away from SIRT1 [134,135]. This shift functionally disconnects metabolic sensing from nuclear and mitochondrial control, contributing to impaired stress adaptation and reduced transcriptional robustness in aging hepatocytes [136,137,138].

As an NAD^+^-dependent deacetylase, SIRT1 plays a central role in maintaining epigenetic stability in the liver. By deacetylating histones and chromatin-associated proteins, SIRT1 promotes heterochromatin integrity and restrains age-associated increases in transcriptional noise [139,140]. Declining SIRT1 activity has been linked to erosion of repressive chromatin architecture, including reduced H3K9me3 deposition, altered coordination of DNA methylation patterns, and loss of transcriptional fidelity [141,142]. Together, these changes compromise hepatocellular identity and increase vulnerability to metabolic and stress-induced dysfunction [136,137,138].

AMPK operates in close functional reciprocity with SIRT1 as a primary cellular energy sensor. In young hepatocytes, reciprocal activation between AMPK and SIRT1 enables rapid and coordinated responses to energetic stress [143,144]. Aging progressively disrupts this reinforcing circuitry, resulting in blunted AMPK responsiveness and attenuation of downstream adaptive programs [7,145]. One key consequence is impaired mitochondrial quality control: reduced SIRT1–AMPK signaling limits mitophagy, allowing dysfunctional mitochondria to accumulate [146]. These damaged organelles generate excess mitochondrial ROS, which can activate cytosolic inflammatory sensors such as the NLRP3 inflammasome, thereby contributing to chronic low-grade inflammation characteristic of hepatic inflammaging [138,147,148].

The transcriptional output of this energy-sensing axis is coordinated by PGC-1α, a master regulator of mitochondrial–nuclear communication [149]. Full transcriptional activity of PGC-1α requires phosphorylation by AMPK and deacetylation by SIRT1 [130,131]. Age-associated disruption of this dual control favors retention of PGC-1α in a transcriptionally constrained state, leading to reduced mitochondrial biogenesis, impaired antioxidant defenses, and persistent bioenergetic insufficiency [150,151]. This transcriptional stagnation reinforces metabolic inflexibility and heightens stress susceptibility in aging hepatocytes [152].

In parallel, aging suppresses signaling through peroxisome proliferator-activated receptor α (PPARα), a nuclear receptor essential for hepatic fatty acid β-oxidation [152,153,154,155]. Attenuated PPARα activity limits lipid catabolic capacity and reduces induction of fibroblast growth factor 21 (FGF21), a hepatokine that coordinates systemic lipid and energy metabolism [156,157,158]. This imbalance favors lipid accumulation and lipotoxic stress, accelerating progression toward hepatic steatosis and further amplifying mitochondrial dysfunction and pro-inflammatory signaling [156,157,158].

**Figure 4:**
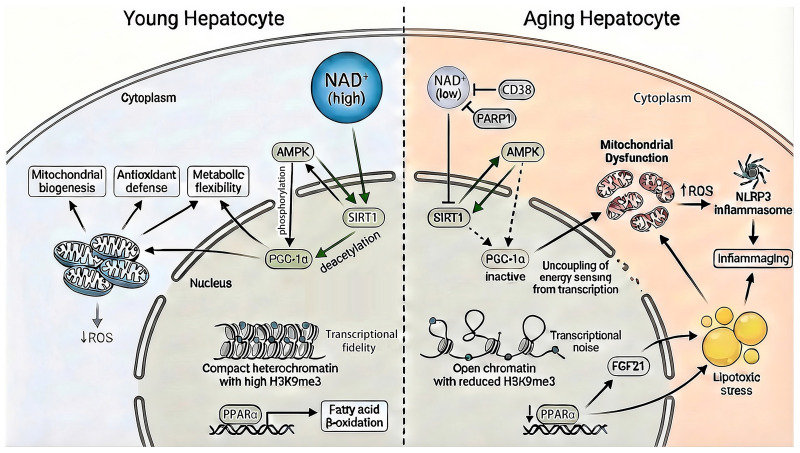
Metabolic decline and epigenetic drift in the aging hepatocyte. Age–associated NAD^+^ depletion driven by NAD^+^–consuming enzymes like CD38 and PARP1 disrupts SIRT1–AMPK–PGC-1α signaling, uncoupling energy sensing from transcriptional and epigenetic regulation. This collapse promotes mitochondrial dysfunction and increased reactive oxygen species (ROS), inflammatory signaling via NLRP3, reduced H3K9me3 and loss of transcriptional fidelity, and lower PPARα leading to lipotoxic stress in aging hepatocytes.

Collectively, NAD^+^ depletion, collapse of SIRT1–AMPK–PGC-1α signaling, and attenuation of PPARα activity converge to drive metabolic exhaustion, mitochondrial decay, and epigenetic drift in the aging liver (Figure 4). Importantly, this coordinated failure creates a therapeutic window for marine-derived bioactives. Rather than functioning as passive antioxidants, select marine carotenoids, polysaccharides, and polyphenols target the metabolic–epigenetic interface that underlies hepatic resilience and homeostatic plasticity.

### 3.2. Carotenoids

Marine carotenoids constitute a structurally diverse class of tetraterpenoids whose biological activity extends beyond classical antioxidant function. Fucoxanthin, astaxanthin, and siphonaxanthin modulate conserved energy-sensing and transcriptional pathways in hepatocytes through distinct molecular entry points, yielding complementary effects on hepatic metabolic homeostasis.

Fucoxanthin primarily acts upstream as an activator of AMPK. In aged or metabolically stressed hepatocytes, where AMPK responsiveness is attenuated, fucoxanthin restores phosphorylation of the AMPKα subunit, re-establishing energy-dependent control over lipid metabolism and mitochondrial maintenance [74,75,77]. AMPK activation suppresses de novo lipogenesis while promoting fatty acid β-oxidation, thereby alleviating metabolic overload [159]. Enhanced AMPK signaling also increases NAD^+^ availability, indirectly supporting SIRT1 activity and re-coupling cellular energy status with epigenetic and transcriptional regulation [74,77]. Through this integrated mechanism, fucoxanthin also stabilizes PGC-1α-dependent mitochondrial programs and improves metabolic flexibility [74,77].

Astaxanthin engages hepatic energy regulation through up-regulation of FGF21 and its downstream effector PGC-1α, key drivers of mitochondrial biogenesis, oxidative metabolism, and lipid catabolism [160]. In hepatocytes, activation of this axis enhances mitochondrial respiratory capacity, reduces lipid accumulation, and mitigates oxidative stress, collectively improving metabolic resilience [160].

Siphonaxanthin (3,3′,19-trihydroxy-7,8-dihydro-β,ε-caroten-8-one) is predominantly derived from green algae and possesses unique hydroxyl and keto substituents that enhance membrane partitioning and bioactivity [161,162] (Figure 3d). Following oral intake, native siphonaxanthin undergoes a multi-stage biotransformation: initial dehydrogenation occurs within the intestinal epithelium, followed by further hepatic processing into a bis-dehydro-metabolite that, along with other active derivatives, preferentially accumulates in the liver and adipose tissue [163]. In hepatocytes, siphonaxanthin suppresses liver X receptor α (LXRα) activation and downstream sterol regulatory element-binding protein-1c (SREBP-1c), reducing transcription of lipogenic genes and expression of fatty acid uptake proteins such as CD36 and fatty acid-binding protein-1 (FABP1) [164]. In preadipocytes and diabetic KK-Ay mice, siphonaxanthin suppresses adipogenesis and fat mass expansion by downregulating key adipogenic and lipogenic genes while enhancing fatty acid oxidation, supporting its role in reducing lipid accumulation [165].

Together, fucoxanthin and astaxanthin reinforce functional coupling within the AMPK–SIRT1–PGC-1α axis, while siphonaxanthin provides complementary regulation of lipid handling through suppression of lipogenesis by inhibiting LXRα (Table 2). Through convergent but non-redundant mechanisms, marine carotenoids support hepatic and systemic metabolic coordination relevant to liver aging.

### 3.3. Polysaccharides

While fucoidan’s antioxidant and anti-inflammatory properties were addressed in Section 2.4, its biological activity extends upstream into metabolic pathways governing hepatic resilience. Rather than acting solely as a cytoprotective scaffold, fucoidan modulates lipid handling and mitochondrial integrity in metabolically stressed hepatocytes.

In diet-induced metabolic stress models, fucoidan significantly reduced hepatic lipid accumulation through a SIRT1-dependent mechanism. Fucoidan supplementation increased hepatic SIRT1 expression, and genetic knockdown of SIRT1 abolished its lipid-lowering effects [166]. In parallel, fucoidan reversed high-fat diet (HFD)-induced suppression of PPARα nuclear localization and lipolytic gene expression, while attenuating PPARγ-associated lipogenic signaling, thereby restoring a lipid oxidative transcriptional balance [166]. Although demonstrated in a non-mammalian liver model, these findings support functional crosstalk between SIRT1 and PPARα in mediating fucoidan’s metabolic effects.

Fucoidan also preserves mitochondrial function in the liver. In rodent models of ethanol- or diet-induced liver injury, fucoidan attenuated mitochondrial dysfunction and reduced accumulation of damaged organelles, consistent with improved mitochondrial quality control under metabolic stress [167]. While direct epigenetic mechanisms were not assessed, maintenance of mitochondrial integrity is a key determinant of NAD^+^ availability and redox balance, both of which regulate epigenetic enzyme activity in hepatocytes. Outside the liver, fucoidan suppresses NLRP3 inflammasome activation via enhancement of selective autophagy in non-hepatic systems [168], supporting a broader link between organelle quality control and restraint of inflammatory amplification relevant to hepatic aging.

Overall, current evidence positions fucoidan as a systems-level metabolic regulator that stabilizes hepatic lipid metabolism through SIRT1-dependent modulation of PPARα signaling and preservation of mitochondrial integrity, indirectly reinforcing the metabolic–epigenetic axis underlying hepatocellular resilience (Table 2).

### 3.4. Polyphenols

Marine polyphenols, particularly phlorotannins derived from brown algae, represent a structurally distinct class of bioactives with emerging relevance to metabolic resilience. Among these, dieckol—a hexameric phloroglucinol derivative from *Ecklonia cava*—has been extensively characterized for its antioxidant capacity and ability to modulate intracellular metabolic signaling pathways [169]. Its high density of hydroxyl groups confers strong redox-buffering properties and facilitates interaction with redox-sensitive signaling nodes [169] (Figure 3d). As a high-molecular-weight marine phlorotannin, the absolute systemic bioavailability of intact dieckol is inherently restricted by poor gastrointestinal absorption. However, following oral intake, unabsorbed dieckol undergoes extensive biotransformation by the intestinal microbiota to generate smaller, highly bioavailable phenolic metabolites, such as phloroglucinol [170,171]. These active microbial metabolites readily enter portal circulation and partition into the hepatic parenchyma [170,171]. Upon entering the liver, these microbial derivatives undergo targeted Phase II biotransformation, including extensive glucuronidation and sulfation, which modulates their local residence time and biological activity [170].

Multiple cellular and animal models demonstrate that dieckol exerts intracellular effects on kinase signaling and mitochondrial function. Dieckol and dieckol-rich *Ecklonia cava* extracts consistently activate AMPK, leading to reduced lipid accumulation, attenuation of oxidative stress, and improved mitochondrial function under metabolic challenge [172,173,174]. These effects support a role for dieckol in restoring metabolic flexibility rather than functioning solely as a passive antioxidant.

Direct evidence for epigenetic modulation by dieckol remains limited. However, AMPK activation provides an indirect mechanistic link to transcriptional and epigenetic regulation through AMPK–SIRT1–PGC-1α crosstalk. In vivo studies report increased hepatic SIRT1 expression following supplementation with *Ecklonia cava* polyphenol extracts [173], although direct enzymatic activation of SIRT1 by dieckol has not been demonstrated. Through this metabolic–transcriptional coupling, dieckol may promote mitochondrial biogenesis, antioxidant defense, and restraint of stress-associated inflammatory signaling rather than acting as a classical chromatin-modifying agent.

Collectively, current evidence positions dieckol as a metabolically active marine polyphenol that reinforces energy-sensing and redox homeostasis pathways central to hepatic resilience (Table 2). By modulating AMPK-linked signaling with downstream effects on mitochondrial function and stress adaptation, dieckol complements carotenoids and polysaccharides in targeting the metabolic–epigenetic interface disrupted during hepatic aging.

## 4. Translational Opportunities and Challenges of Marine Bioactives in Liver Rejuvenation

The transition of marine bioactives from mechanistic promise to clinically actionable interventions represents a critical next step in hepatic rejuvenation research. While the pathways outlined in Section 2 and Section 3 establish a strong biological rationale, successful translation requires demonstration of in vivo efficacy, favorable pharmacokinetics, and solutions to age-specific delivery challenges. To date, no late-phase clinical trials have been conducted, and clinical application remains largely exploratory. Nonetheless, a growing body of preclinical evidence supports the therapeutic plausibility of marine-derived compounds as modulators of age-associated liver dysfunction.

### 4.1. Preclinical Evidence: Efficacy in Animal Models of Liver Dysfunction

Marine bioactives discussed in previous sections have been evaluated across a spectrum of in vivo models that recapitulate clinically relevant features of hepatic aging, including steatosis, chronic inflammation, insulin resistance, and fibrotic remodeling. Across these models, improvements in histological, biochemical, and functional endpoints indicate that pathway-level modulation translates into meaningful attenuation of liver pathology.

Fucoxanthin has shown beneficial effects in diet-induced and genetic models of metabolic liver disease. In diabetic (db/db) mice and HFD models, fucoxanthin reduces hepatic triglyceride accumulation, normalizes liver weight, and improves serum transaminase levels, indicating attenuation of steatosis and hepatocellular injury [175,176,177]. These hepatic improvements are accompanied by enhanced systemic glucose tolerance, lipid profiles, and insulin sensitivity—endpoints relevant to MAFLD pathophysiology [176,177].

Astaxanthin robustly reverses fatty liver phenotypes in both genetic (ob/ob) and HFD-fed models, producing marked reductions in hepatic lipid droplet accumulation, oxidative stress, and inflammatory infiltrates [160,178,179]. These effects translate into attenuation of histopathological disease features, including lobular inflammation and hepatocellular ballooning [160,179]. Consistent with MAFLD pathophysiology, astaxanthin lowers hepatic triglyceride and cholesterol content while normalizing systemic metabolic markers such as fasting blood glucose and insulin resistance [179]. Protective effects extend to more advanced disease states, where astaxanthin attenuates collagen deposition and fibrotic progression in carbon tetrachloride (CCl_4_)-induced liver injury models [180]. Collectively, findings across metabolic and toxic liver injury paradigms position astaxanthin as a strong candidate for early-stage metabolic liver disease intervention.

Although less extensively studied, siphonaxanthin has demonstrated activity in murine models of obesity-associated metabolic stress. In ob/ob mice maintained on a HFD, siphonaxanthin treatment is associated with improvements in biochemical and oxidative stress-related endpoints, including reductions in plasma alanine aminotransferase (ALT), hepatic lipid peroxidation, and renal protein carbonyl content [181].

Fucoidan has been evaluated across multiple experimental models of liver injury, with particularly strong evidence in fibrosis-prone and toxin-induced settings. In CCl_4_-induced liver injury, fucoidan attenuates hepatocellular damage, reduces collagen accumulation, and limits hepatic stellate cell activation [182,183]. These histological improvements are accompanied by consistent reductions in serum markers of liver injury, including aminotransferases [182,183]. In metabolic liver disease models, fucoidan decreases hepatic lipid accumulation and inflammatory cytokine expression while improving systemic insulin sensitivity and glycemic control [184]. Together, these findings position fucoidan among the more translationally mature marine bioactives, with demonstrated efficacy across steatosis, inflammation, and fibrogenic remodeling. Notably, co-administration with fucoxanthin has yielded additive improvements in metabolic parameters and liver function markers [185,186].

Dieckol has demonstrated hepatoprotective activity across multiple preclinical models of liver injury. In CCl_4_-induced liver murine models, dieckol attenuates hepatocellular damage and inflammatory degeneration, accompanied by improvements in serum liver enzyme levels [187]. In metabolically stressed mice, dieckol administration significantly lowers hepatic and systemic inflammatory burden while limiting inflammatory cell infiltration [188,189]. Histological analyses consistently show reduced inflammatory lesions and improved hepatic morphology [188,189].

Bromophenol derivatives and marine-derived peptide fractions, including peptides from oyster and krill sources, demonstrate hepatoprotective effects across multiple experimental models of liver injury. In HFD-induced and toxin-mediated liver injury systems, certain marine peptide preparations attenuate elevations in serum aminotransferases and other markers of hepatocellular damage, and limit histopathological evidence of necrosis and inflammation [190,191]. In ethanol-fed mouse models of alcoholic liver disease, oyster-derived peptides and other marine peptide fractions consistently decrease serum ALT and aspartate aminotransferase (AST) levels, reduce hepatic triglyceride accumulation, and attenuate inflammatory cytokine and lipid peroxidation levels, with accompanying improvements in hepatic histology [192]. Bromophenol derivatives, while less extensively studied in classical liver injury models, significantly reduce plasma glucose, serum triglycerides, and total cholesterol in diabetic mice, supporting their potential relevance in metabolic dysfunction states [193].

Overall, these in vivo studies demonstrate that marine bioactives can improve clinically relevant endpoints—including steatosis, inflammation, and fibrosis—across diverse models of liver dysfunction (Table 3). These findings provide strong proof-of-concept that pathway-targeted interventions can preserve liver structure and function in the context of aging and metabolic stress, justifying continued investment in advanced formulation development and extended studies in aged animal models.

### 4.2. Translational Outlook and Early Human Studies

Building on robust preclinical findings, the translation of marine bioactives into human interventions is an active area of investigation. While large-scale clinical trials are still needed, early-phase studies are beginning to validate their metabolic and hepatoprotective effects. For example, a double-blind, randomized, placebo-controlled trial showed that 24 weeks of astaxanthin supplementation (12 mg/day) in individuals with prediabetes and dyslipidemia significantly lowered total cholesterol and LDL, reduced circulating cardiovascular risk markers (fetuin-A, fibrinogen, L-selectin), and improved trends in fasting insulin action [194]. Similarly, fucoxanthin has been evaluated in a 12-week, double-blind, placebo-controlled trial (12 mg/day) in patients with metabolic syndrome, demonstrating reductions in body weight, waist circumference, blood pressure, and serum triglycerides, along with improvements in both first-phase and total insulin secretion [195].

Marine bioactives have also shown preliminary safety and efficacy in chronic viral liver conditions. In an open-label pilot study, oral fucoidan (0.83 g/day) administered for 12 months to patients with chronic HCV infection, including those with cirrhosis, significantly reduced viral loads and trended toward normalization of serum ALT without serious adverse effects [196]. Together, these early human studies suggest that the molecular pathways targeted by marine bioactives are therapeutically accessible and well-tolerated, reinforcing their potential in proactive liver rejuvenation as well as systemic metabolic health.

### 4.3. Translatability and Delivery: Leveraging the Gut–Liver Axis

**Figure 5:**
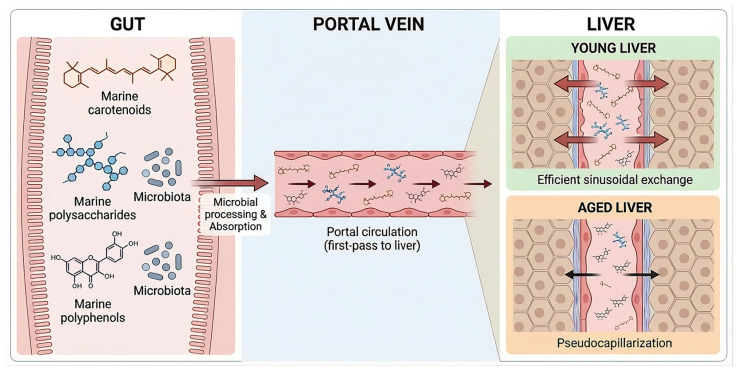
Gut–liver axis and age-dependent hepatic delivery of marine bioactives. Orally administered marine compounds and their microbiota-derived metabolites reach the liver via portal circulation, conferring a first-pass targeting advantage. Aging-associated sinusoidal pseudocapillarization may reduce hepatic delivery, particularly for high-molecular-weight molecules.

A key translational advantage of marine bioactives lies in their alignment with the gut–liver axis. Most compounds discussed herein are orally administered and undergo first-pass exposure to the liver via portal circulation, favoring hepatic targeting without the need for invasive delivery strategies [45,197,198] (Figure 5). Following intestinal absorption, marine carotenoids and their metabolites—such as fucoxanthinol derived from fucoxanthin—enter the portal vein and reach the liver at relatively high concentrations prior to systemic dilution [70] (Figure 5). This pharmacokinetic feature enhances local efficacy while potentially limiting off-target exposure.

The gut microbiota further acts as a metabolic amplifier for several marine compounds. Polysaccharides such as fucoidan function as prebiotics, indirectly shaping microbial composition and metabolite output [199,200], while complex polyphenols undergo microbial biotransformation into smaller, more bioavailable phenolic acids [201,202] (Figure 5). These microbiota-derived metabolites may account for a substantial proportion of observed hepatic effects, underscoring the importance of host–microbe interactions in therapeutic responsiveness [203].

An additional translational advantage is the favorable safety and tolerability profile observed for many marine bioactives. Long-standing dietary exposure in human populations provides a foundational safety signal that may facilitate early-phase clinical testing and dose escalation studies [204,205]. Moreover, the pleiotropic, pathway-modulating nature of these compounds aligns well with the multifactorial biology of liver aging, offering a systems-level alternative to single-target pharmacological approaches.

### 4.4. Translational Barriers and Unresolved Challenges

Despite these advantages, several biological, technological, and regulatory barriers continue to constrain clinical implementation of marine bioactives. Poor aqueous solubility and oxidative instability—particularly for carotenoids such as astaxanthin—remain significant formulation challenges [206,207]. Advanced delivery strategies, including nano-lipid carriers, self-emulsifying drug delivery systems, and micellar encapsulation, are being actively developed to enhance intestinal absorption, preserve bioactivity, and improve dose consistency [207,208,209,210,211,212]. Such approaches will be essential for transitioning marine bioactives from nutraceutical-grade preparations to clinically reliable interventions. In parallel, deeper mechanistic characterization, improved purification pipelines, and scalable synthesis strategies will be required to ensure consistent bioactivity and clinical-grade production.

A defining feature of the aging liver is pseudocapillarization of liver sinusoidal endothelial cells, characterized by loss of fenestrae and progressive deposition of extracellular matrix components along the sinusoidal wall [213,214]. Rather than increasing permeability, this structural remodeling reduces sinusoidal exchange efficiency, limiting the transfer of lipoproteins, metabolites, and xenobiotics from the circulation to hepatocytes [213,214] (Figure 5). Consequently, the delivery and efficacy of large molecules may be substantially impaired in aged or diseased livers [215,216] (Figure 5). Thus, bioavailability and efficacy in young animal models may overestimate therapeutic performance in aging humans.

Standardization and regulatory compliance remain significant challenges for marine bioactives. Although many are classified as generally recognized as safe (GRAS), variability in molecular composition—driven by species differences, harvest conditions, and extraction methods—can limit reproducibility across studies [217,218]. Contamination with heavy metals or marine toxins remains a non-trivial concern [219]. While preclinical and early human studies suggest a favorable safety profile for compounds such as fucoxanthin, astaxanthin, fucoidan, and dieckol, supra-physiological doses have been associated with gastrointestinal disturbances, mild hepatotoxicity, and alterations in lipid metabolism [220,221,222]. For instance, the anticoagulant activity of high-dose fucoidan underscores the need for careful dose optimization and safety monitoring [223,224]. Establishing the therapeutic window and maximum tolerated dose for each bioactive will therefore be essential, particularly if their clinical use extends beyond nutritional supplementation toward disease-modifying or liver-rejuvenative applications. Beyond dose-dependent toxicity, the pleiotropic nature of these compounds raises the potential for off-target effects, particularly when modulating conserved metabolic, inflammatory, and epigenetic pathways. Possible interactions with drug-metabolizing enzymes and concomitant therapies remain insufficiently characterized, and long-term safety data in humans, especially at pharmacological doses, are still limited. Addressing these challenges will be essential to define therapeutic windows and meet regulatory standards for clinical translation.

A further limitation is the heavy reliance on acute or subacute injury models in young animals, which inadequately capture the slow, cumulative epigenetic drift, mitochondrial dysfunction, and immune remodeling that define human liver aging. Long-term intervention studies in aged animal models and rigorously designed human trials remain scarce. Bridging this translational gap will require chronic dosing paradigms, age-appropriate pharmacokinetic assessments, and biomarker strategies capable of capturing functional rejuvenation rather than short-term injury resolution alone.

## 5. Marine Chemodiversity and Cellular Reprogramming: Conceptual Links and Future Directions

Aging of the liver is increasingly recognized as an active, maladaptive remodeling process rather than a passive accumulation of damage. One emerging hallmark of this process is mesenchymal drift, in which hepatocytes progressively acquire mesenchymal-like transcriptional and epigenetic features under chronic stress [225]. This shift is characterized by partial activation of fibrogenic, inflammatory, and extracellular matrix-associated gene programs within hepatocytes themselves, leading to impaired metabolic function, reduced regenerative capacity, and heightened susceptibility to fibrosis and carcinogenesis [225]. Importantly, mesenchymal drift reflects erosion of lineage fidelity rather than irreversible cell fate conversion, suggesting that hepatocellular identity could, in principle, be reinforced or restored [225].

Within this context, advances in aging biology have demonstrated that age-associated cellular states are amenable to partial reversal. Partial cellular reprogramming, achieved through transient induction of Yamanaka factors (OCT4, SOX2, KLF4, and c-MYC; OSKM), has been shown to reset epigenetic aging markers, restore youthful transcriptional programs, and improve mitochondrial and regenerative function without complete dedifferentiation when applied in a tightly controlled manner [36,37,38,39]. In experimental hepatic models, intermittent or short-term OSKM expression can ameliorate several molecular hallmarks of aging and reduce fibrotic features, underscoring the intrinsic plasticity of hepatocytes [36,37]. However, these benefits arise not from reversion to pluripotency, but from selective reactivation of gene expression profiles associated with youthful chromatin states and metabolic networks.

Despite these promising findings, prolonged or excessive OSKM induction poses substantial risks, including loss of tissue integrity, organ failure, teratoma formation, and premature mortality in vivo [36,226,227]. These limitations have prompted the search for lineage-preserving alternatives that restore youthful function without destabilizing cell identity. One such strategy involves modulation of pioneer transcription factors that define and maintain hepatocyte fate. In the liver, factors such as HNF4α act as chromatin-organizing regulators that sustain hepatocyte-specific gene networks, metabolic homeostasis, and transcriptional stability [228,229]. Age-associated decline in pioneer factor activity contributes to identity erosion and mesenchymal drift, while restoration or stabilization of these factors has shown promise in preclinical models in reinforcing epithelial gene dominance and suppressing maladaptive programs without inducing dedifferentiation [230,231]. This approach highlights a tissue-specific route to potential rejuvenation that minimizes the oncogenic and developmental risks associated with broad reprogramming.

In parallel, chemical reprogramming strategies have emerged as a non-genetic modality for inducing partial rejuvenation in vitro and in animal models. Small-molecule cocktails targeting conserved aging pathways—including epigenetic modifiers, TGF-β signaling, mitochondrial function, AMPK–mTOR balance, and sirtuin activity—can partially recapitulate aspects of genetic reprogramming [40,41]. In hepatic contexts, such interventions have been shown in preclinical studies to improve metabolic competence, attenuate senescence-associated transcriptional signatures, and stabilize hepatocyte identity in aged or diseased models [232]. These findings reinforce the concept that liver rejuvenation could theoretically be achieved through coordinated modulation of chromatin state, metabolism, and stress resilience, rather than wholesale cell fate conversion. However, chemical reprogramming faces its own limitations, including incomplete efficacy, narrow therapeutic windows, and challenges in maintaining durable, tissue-specific effects [233]. This has catalyzed interest in adjunctive compounds capable of lowering the energetic and epigenetic barriers to rejuvenation while preserving cellular identity.

Within this framework, marine-derived bioactive compounds emerge as compelling theoretical candidates to interface with experimental reprogramming-based strategies. As detailed throughout this review, marine natural products target multiple biological axes implicated in hepatic aging, including oxidative stress, mitochondrial quality control, inflammatory signaling, fibrotic remodeling, and nutrient sensing. In preclinical models of chronic liver disease—conditions that closely mirror age-associated hepatic decline—marine polysaccharides, carotenoids, and polyphenols have demonstrated the capacity to suppress senescence-associated signaling, attenuate pro-fibrotic transcriptional programs, and restore metabolic flexibility.

It is hypothesized that rather than acting as direct reprogramming agents, marine bioactives may function as enabling or stabilizing components within chemical reprogramming networks. By improving redox balance, preserving mitochondrial efficiency, and dampening inflammation, these compounds may create a permissive intracellular environment that could enhance the safety, specificity, and durability of partial reprogramming interventions. Moreover, by supporting chromatin stability and hepatocyte-specific transcriptional programs, marine bioactives may indirectly counteract mesenchymal drift without inducing dedifferentiation.

From this perspective, marine chemodiversity represents not merely a source of hepatoprotective agents, but a largely untapped reservoir of rejuvenation-adjacent modulators. The structural novelty of marine metabolites—shaped by extreme evolutionary pressures—expands accessible chemical space beyond that of most terrestrial compounds. For example, halogenation, as seen in marine bromophenols, can confer binding affinities and interaction geometries that are rarely achievable with non-halogenated natural products. Systematic screening of marine libraries for their ability to synergize with experimental partial reprogramming, pioneer factor stabilization, or chemical rejuvenation strategies represents a rational and testable direction for future research.

Beyond reprogramming-centered frameworks, several emerging dimensions of liver aging further refine the landscape of therapeutic intervention. Cellular senescence is increasingly recognized as a driver of hepatic dysfunction, and the development of senolytic and senomorphic strategies offers a complementary approach to rejuvenation by selectively eliminating or functionally modulating senescent hepatocytes and non-parenchymal cells [9,234]. In parallel, spatial and single-cell transcriptomic analyses have revealed pronounced intrahepatic heterogeneity during aging, with zonation-dependent vulnerabilities that differentially affect metabolic, inflammatory, and fibrotic pathways [235,236]. Additional mechanisms such as ferroptosis have been implicated in age-associated liver injury and may intersect with mitochondrial dysfunction and redox imbalance [237,238]. At the systems level, the emergence of liver-specific epigenetic clocks and multi-omics signatures is enabling increasingly precise quantification of biological aging and therapeutic response [235,239,240]. Together, these advances highlight the need for integrative strategies that account for cellular state, spatial context, and molecular heterogeneity. Within this evolving framework, marine bioactives—through their pleiotropic effects on redox homeostasis, inflammation, and metabolic regulation—are well positioned to interface with emerging senescence-modulating therapies, ferroptosis regulation, and multi-omics-guided precision interventions, further expanding their potential relevance in next-generation liver rejuvenation paradigms.

Collectively, this integrative framework reframes marine liver research within a conceptually rejuvenation-oriented paradigm. By shifting focus from damage mitigation toward the potential restoration of youthful cellular states and lineage fidelity, the convergence of marine pharmacology and cellular reprogramming biology offers a coherent theoretical strategy for advancing liver rejuvenation. While extensive experimental and clinical validation is still required, this intersection provides a biologically grounded foundation for positioning marine natural products within the evolving landscape of regenerative medicine.

## 6. Conclusions

Aging progressively compromises liver function through interconnected metabolic, mitochondrial, epigenetic, and inflammatory alterations that erode hepatocellular identity and regenerative capacity. Marine-derived bioactive compounds engage many of these same regulatory nodes, modulating energy sensing, redox balance, mitochondrial quality control, and chromatin-associated pathways that collectively govern cellular resilience in the aging liver. Across diverse chemical classes, marine bioactives have demonstrated in preclinical models the capacity to influence pathways associated with cellular state rather than merely attenuating damage accumulation. This positions them as compelling candidates within a conceptually rejuvenation-oriented framework rather than strictly a disease suppression paradigm.

By organizing existing evidence around aging hallmarks and regulatory networks, this review provides a conceptual framework for researchers in liver biology, aging, and bioactive therapeutics to systematically evaluate marine compounds as modulators of hepatic resilience. In particular, the theoretical integration of marine chemodiversity with emerging experimental approaches such as partial cellular reprogramming highlights new opportunities for exploring combinatorial strategies that require precise metabolic and epigenetic control.

Future efforts should prioritize the identification and screening of novel marine compounds targeting conserved aging pathways, alongside rigorous evaluation of their pharmacokinetics, bioavailability, and tissue-specific effects. Advancing translation will require improved formulation and standardization, as well as validation in clinically relevant models of liver aging and age-associated diseases such as MASH and fibrosis. In parallel, deeper investigation into safety, long-term effects, and potential combinatorial use with emerging rejuvenation strategies will be essential to define their true therapeutic potential and confirm whether preclinical pathway modulation can effectively translate into organ-level rejuvenation.

Collectively, marine chemodiversity represents a largely untapped resource for shaping next-generation liver-directed interventions that aim to preserve or potentially restore function by targeting aging vulnerabilities as a modifiable driver of disease.

## Figures and Tables

**Table 1:** Summary of structurally diverse marine-derived bioactive compounds, their primary molecular targets, and their functional impact on the redox–senescence axis of aging hepatocytes.

Marine Bioactive	Compound Class	Primary Molecular Targets	Impacts on Oxidative Stress & Senescence
Fucoxanthin	Carotenoid	Activates NRF2/ARE and SIRT1; activates AMPK; inhibits TLR4/NF-κB	Mitigates ROS and genomic damage; improves mitochondrial quality control; suppresses SASP
Astaxanthin	Carotenoid	Intercepts ROS in lipid bilayers; engages NRF2/HO-1; inhibits NF-κB and MAPK	Stabilizes mitochondrial membranes; inhibits ferroptosis; prevents irreversible cell-cycle arrest (p16^INK4A^)
Fucoidan	Polysaccharide	Polyanionic mimetic; stabilizes nuclear NRF2; inhibits TGF-β1/Smad	Sustains redox balance via HO-1/glutathione; limits p21^WAF1^ and p16^INK4A^; restrains pro-senescent fibrotic niches
Bromophenol Derivatives	Bromophenol	Activates NRF2 by disrupting KEAP1 repression; inhibits PTP1B	Induces cytoprotective genes; preserves metabolic flexibility and insulin sensitivity
Asp–Leu–Val–Lys–Val–Glu–Ala, Ala–Tyr (AY), QDYD, ARW, YPAGP	Bioactive Peptides	Activates AMPK/NRF2 axis	Stabilizes mitochondria; promotes fatty acid β-oxidation; enhances endogenous antioxidant enzymes

**Table 2:** Mechanistic summary of marine-derived bioactives targeting the metabolic–epigenetic interface in aging hepatocytes.

Marine Bioactive	Compound Class	Primary Molecular Target (s)	Epigenetic & Metabolic Impact
Fucoxanthin	Carotenoid	AMPK	Restores energy-dependent control over lipid metabolism; increases NAD^+^ availability to support SIRT1 activity
Astaxanthin	Carotenoid	FGF21/PGC-1α	Enhances mitochondrial respiratory capacity and adaptive transcriptional programs; mitigates oxidative stress
Siphonaxanthin	Carotenoid	LXRα	Suppresses lipogenesis via SREBP-1c inhibition; induces fatty acid oxidative gene expression
Fucoidan	Polysaccharide	SIRT1/PPARα	Restores lipid oxidative transcriptional balance; improves mitochondrial quality control and mitophagy
Dieckol	Polyphenol	AMPK/SIRT1	Reinforces energy-sensing and redox homeostasis; promotes mitochondrial biogenesis via AMPK–SIRT1–PGC-1α crosstalk

**Table 3:** Summary of marine-derived bioactive compounds and their therapeutic efficacy across preclinical animal models of liver dysfunction recapitulating key features of age-associated hepatic decline, including metabolic stress, inflammation, and fibrotic remodeling. HFD: high-fat diet. *w*/*w*: weight per weight. PO: oral administration. IV: intravenous. LPS/D-GalN: lipopolysaccharide/D-galactosamine.

Marine Bioactive	Experimental Model (s) (Organism)	Key Biochemical & Histological Improvements	Relevant Clinical Endpoint (s)	Dose (Route)	Duration of Treatment	References
Fucoxanthin	db/db (mice), HFD (mice)	Reduced hepatic triglycerides; normalized liver weight; improved transaminase levels	Steatosis, insulin sensitivity, and glucose tolerance	db/db: 0.2–0.4% (*w*/*w*) HFD: 35–60 mg/kg (PO)	db/db: 6 weeks HFD: 8 weeks	db/db: [177] HFD: [176]
Astaxanthin	ob/ob (mice), HFD (mice), CCl_4_ (rats)	Reduced lipid droplets and inflammatory infiltrates; attenuated ballooning and collagen deposition	Steatosis, inflammation, and fibrogenesis	ob/ob: 0.02% (*w*/*w*) HFD: 0.02% (*w*/*w*) CCl_4_: 10 mg/kg (PO)	ob/ob: 10 weeks HFD: 12 weeks CCl_4_: 2 weeks	ob/ob: [179] HFD: [179] CCl_4_: [180]
Siphonaxanthin	ob/ob (mice) + HFD	Improved transaminase levels; decreased hepatic lipid peroxidation and renal protein carbonyls	Obesity-associated metabolic and oxidative stress	0.016% (*w*/*w*)	43 days	[181]
Fucoidan	CCl_4_ (mice/rats), HFD (mice)	Decreased hepatic stellate cell activation and collagen deposition; reduced inflammatory cytokines	Fibrogenesis, inflammation, and glycemic control	CCl_4_: 50 mg/kg (mice, IV), 100 mg/kg (rats, PO) HFD: 1–5% (*w*/*w*)	CCl_4_: 8 weeks (mice), 2 weeks (rats) HFD: 12 weeks	CCl_4_: [182,183] HFD: [184]
Dieckol	CCl_4_ (mice), HFD (mice)	Improved hepatic morphology; reduced inflammatory cytokines	Inflammation and hepatocellular damage	CCl_4_: 5–25 mg/kg (PO) HFD: 2.5 mg/kg (PO)	CCl_4_: 1 week HFD: 4 weeks	CCl_4_: [187] HFD: [188]
Marine Peptides	HFD (mice), LPS/D-GalN (mice), ethanol (mice)	Improved transaminase levels; reduced hepatic triglycerides and lipid peroxidation	Necrosis, inflammation, and alcoholic liver disease	HFD: 50–250 mg/kg (PO) LPS/D-GalN: 10–50 mg/kg (PO) Ethanol: 100–900 mg/kg (PO)	HFD: 4 weeks LPS/D-GalN: 10 days Ethanol: 3 days	HFD: [190] LPS/D-GalN: [191] Ethanol: [192]
Bromophenols	db/db (mice)	Reduced plasma glucose, serum triglycerides, and total cholesterol	Metabolic dysfunction and dyslipidemia	10–40 mg/kg (PO)	8 weeks	[193]

## Data Availability

No new data were created or analyzed in this study.

## Abbreviations

The following abbreviations are used in this manuscript:

ALT	Alanine Aminotransferase
AMPK	AMP-Activated Protein Kinase
ARE	Antioxidant Response Element
AST	Aspartate Aminotransferase
CCl_4_	Carbon Tetrachloride
FGF21	Fibroblast Growth Factor 21
HFD	High-Fat Diet
HO-1	Heme Oxygenase-1
IV	Intravenous
KEAP1	Kelch-Like ECH-Associated Protein 1
LPS/D-GalN	Lipopolysaccharide/D-galactosamine
MAFLD	Metabolic Dysfunction-Associated Steatotic Liver Disease
MASH	Metabolic Dysfunction-Associated Steatohepatitis
NRF2	Nuclear factor erythroid 2-related factor 2
NQO1	NAD(P)H Quinone Dehydrogenase 1
OSKM	OCT4, SOX2, KLF4, c-MYC
PO	Oral administration
PPARα	Proliferator-Activated Receptor α
ROS	Reactive Oxygen Species
SASP	Senescence-Associated Secretory Phenotype
SOD	Superoxide Dismutase
*w*/*w*	Weight per weight
